# Supplementary material for: Chlamydia buteonis in birds of prey presented to California wildlife rehabilitation facilities
Source: PLoS One. 2021 Oct 13;16(10):e0258500. doi: 10.1371/journal.pone.0258500 (PMC8513822; doi:10.1371/journal.pone.0258500)
Supplement: Attachment — Submitted filename: 2021_PlosOne_Response to Reviewers FINAL.docx [file pone.0258500.s001.docx]

Manuscript PONE-D-21-20941

Response to Reviewers

Dear Dr. Dean,

Thank you for giving us the opportunity to re-submit a revised manuscript of “Chlamydia buteonis in birds of prey presented to California wildlife rehabilitation facilities” for PlosOne. We appreciate yours along with the reviewers’ time and effort spent providing feedback and insightful comments to help improve our manuscript. We have incorporated most of the suggestions made by you and the reviewers. Please see below a point-by-point response to the reviewers’ comments and concerns. Line numbers refer to the revised manuscript file with tracked changes.

**5. Review Comments to the Author**

Reviewer #1: The findings of this study are new and interesting as they appear shortly after publication of the species Chlamydia buteonis. Samples for serology and qPCR were collected from 263 birds of prey, representing 18 species. Chlamydial DNA was found in 4.18 % and anti-chlamydial Abs detected in 3.14 % of tested birds. As could be expected, a serologic response was typically observed in adults. Nevertheless, the inclusion of serology in parallel to DNA testing is an important asset of the present work. The manuscript is concise and well written. Data processing and presentation are adequate.

A few points remain unclear and should be revised.

1. Line 114: Plasma samples were divided into two aliquots and tested using two serologic assays. Please, indicate the chlamydia species of the hawk isolate used in the assay at the IDL.

**Author Response:** Thank you, the species of the hawk isolates used were added.

2. Line151-52:
a) It is unclear from the text whether both positive cultures from SWHA (IDL16-
5840_SH) and RTHA (IDL17_4553_RTH) were genome sequenced or only one. How many sequences will be (or have been) deposited at NCBI?

**Author Response:** Thank you, both positive cultures were sequenced, and this information was added. Two sequences have been deposited at NCBI under the BioProject accession number PRJNA613727.

b) It seems that complete assembly of the raw sequences has not been achieved as 742 clusters are reported. If so, a reason should be given.

**Author Response:** Thank you, the Anvi'o software determined 742 single copy genes that were distinguishable as homologous between all the genomes and determined to be single copy genes in all the genomes; and therefore, used for the phylogenetic analysis. This was not a measure of completeness of any genome, just the number of genes that were used in the phylogenetic analysis. The description of the methods was updated in the manuscript.

3. Lines 157-59: The qPCR assay for C. buteonis used here is probably new and has not been published previously. If so, the authors should include data showing the specificity of that assay. Besides, the protocol of a specific and sensitive assay can be found in reference 10.

**Author Response:** Thank you, we expanded on the flow of the *C. buteonis* identification in the positive samples within the method section. The samples were first analysed using the *C. psittaci* specific qPCR with a melting curve analysis following a previously described method. However, the positive samples from this study showed a different melting curve signature compared to *C. psittaci* positive control samples. Thus, those samples were amplified using *C. buteonis* specific primers described in the method section and then Sanger sequenced to confirm *C. buteonis* identity.

4. Line 207: The authors claim that the clade containing their own C. buteonis isolates is distinctly different from other chlamydial species. However, there is only one more species, C. psittaci, on Fig. 1. To substantiate the statement, at least one more species should appear in the figure, e.g. C. trachomatis as a non-avian Chlamydia sp.

**Author Response:** Thank you, we understand that the conclusion suggested results showing more than 1 species and therefore edited the results section of Figure 1 to state the following “This analysis supported the inclusion of the SWHA and RTHA isolates in the clade containing *C. buteonis* as determined by Laroucau et al, which is a phylogenetically intermediate between *C. psittaci* and *C. abortus.”*

5. In reference 10, only hawks (genus Buteo) were mentioned as known hosts of C. buteonis. The present study revealed new observations on host specificity of this species that should be summarized and included in the text as a major conclusion.

**Author Response:** Thank you. This finding was emphasized in the abstract, discussion and conclusion.

Reviewer #2: The authors present an analysis of detection of chlamydial DNA and/or antibodies in raptors presenting to wildlife rehabilitation centres. The analysis is clear and easy to follow, and the trends apparent related to age, different species are also clearly documented. The sample size of 263 from 18 species is good size, and across several distinct centres. The evidence of increasing serological responses in age and chlamydial DNA associated with disease supports the conclusion of the potential for risk and the prevalence of the organism in these wild populations. The sequence analysis confirming similarity to a recently described c.buteonis adds confidence to the findings. Overall this study is a valid and useful addition to the literature on chlamydia in wildlife.
